# Supplementary material for: HOCl-producing electrochemical bandage is active in murine polymicrobial wound infection
Source: Microbiol Spectr. 2024 Aug 20;12(10):e00626-24. doi: 10.1128/spectrum.00626-24 (PMC11448380; doi:10.1128/spectrum.00626-24)
Supplement: Supplemental material — Guide to scoring of in vivo biofilms from SEM images. [file spectrum.00626-24-s0001.pdf]

Guide to scoring of *in vivo* biofilm from SEM images

Mayo Clinic Infectious Diseases Research Laboratory

In vivo biofilms are scored based on the following criteria:

1. Extracellular polymeric substance (EPS) integrity/abundance
2. Integrity of microbial cells
3. Microbial abundance

EPS integrity/abundance examples: Stringy EPS

High: 3

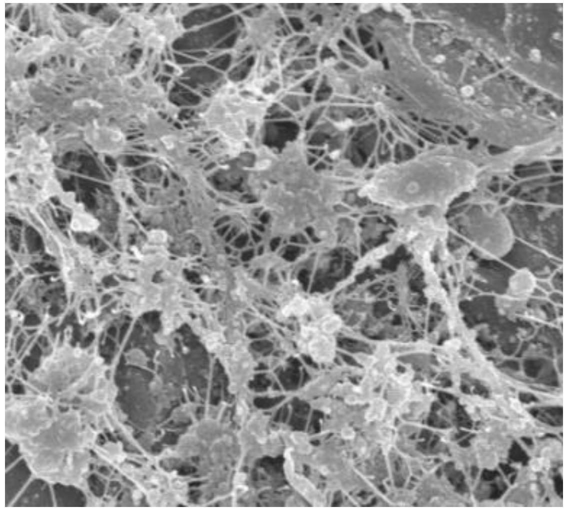

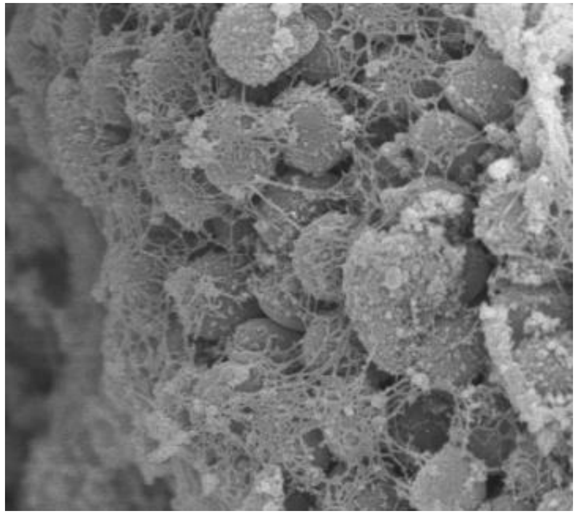

Medium: 2

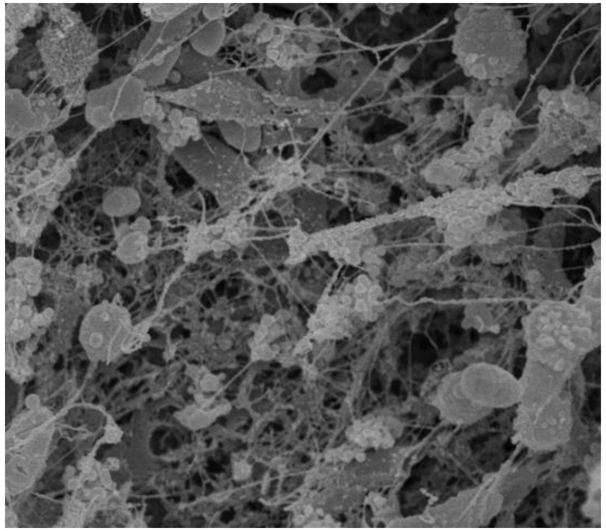

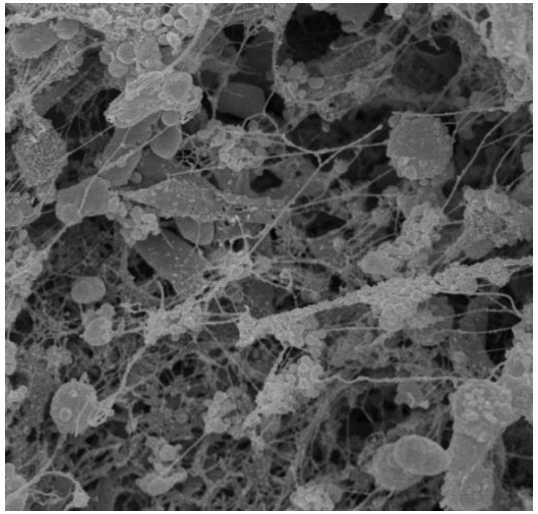

Low: 1

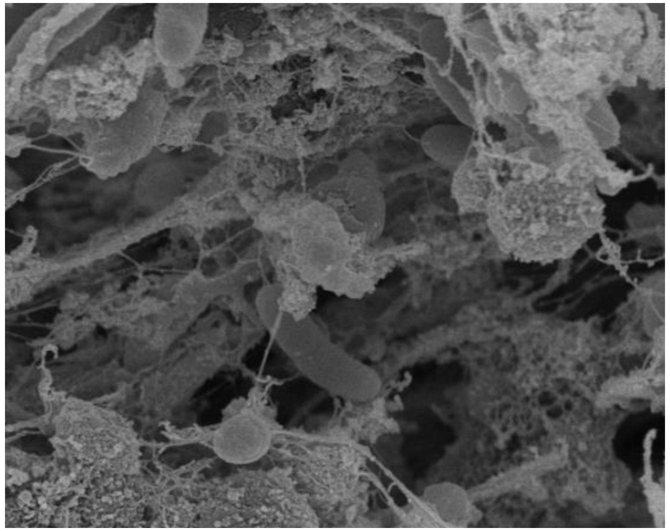

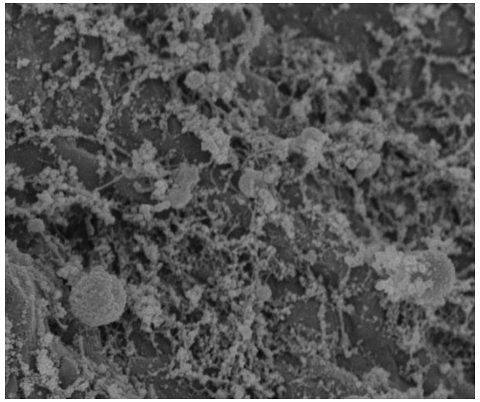

EPS integrity/abundance examples: Granulated EPS

High: 3

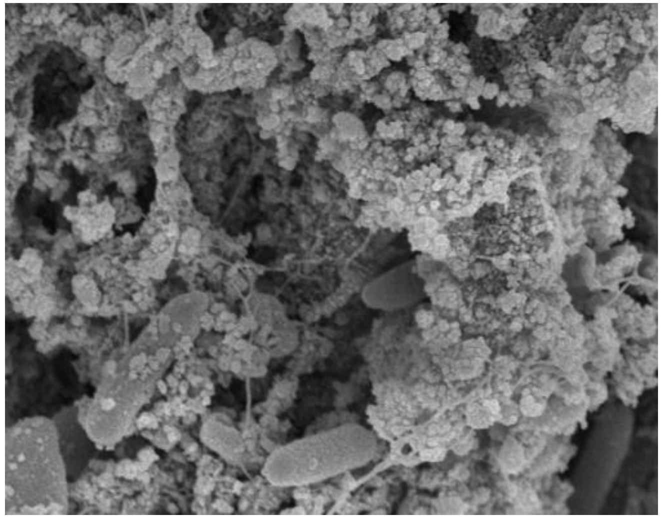

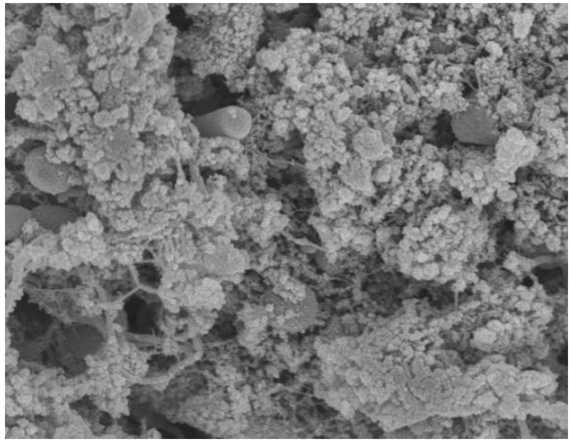

Medium: 2

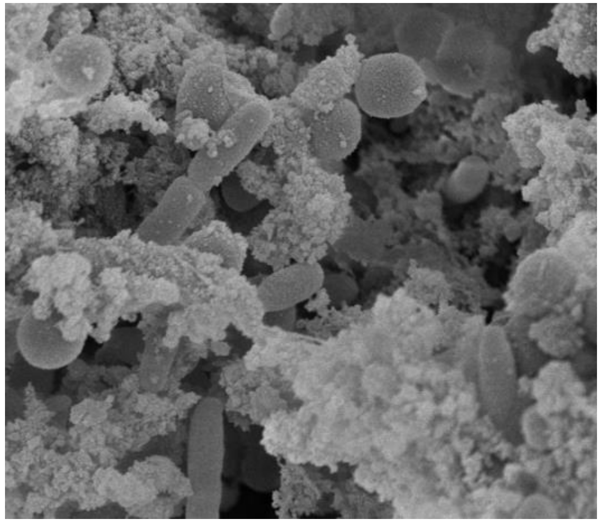

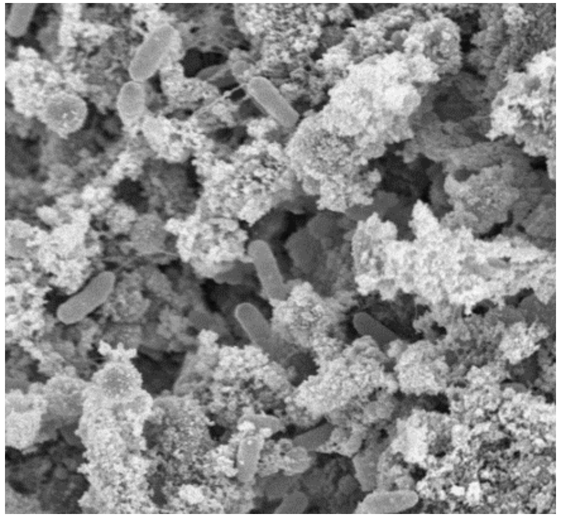

Low: 1

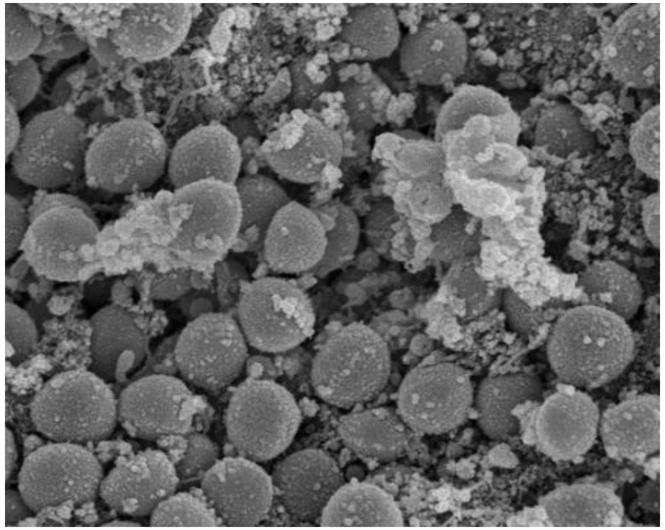

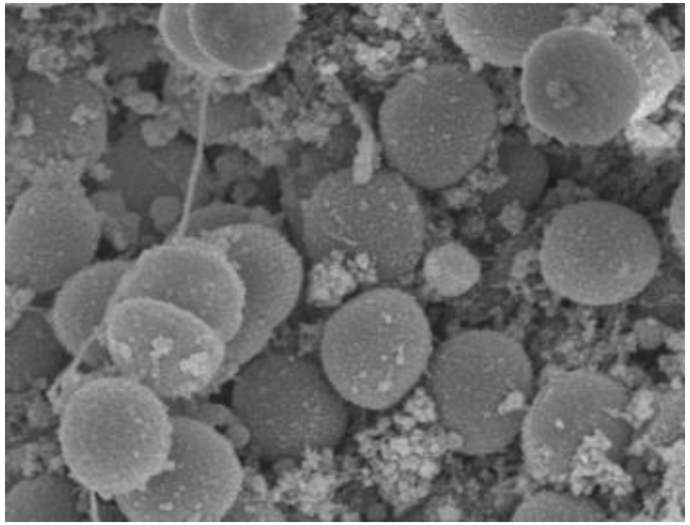

EPS integrity/abundance examples: Combination EPS

High: 3

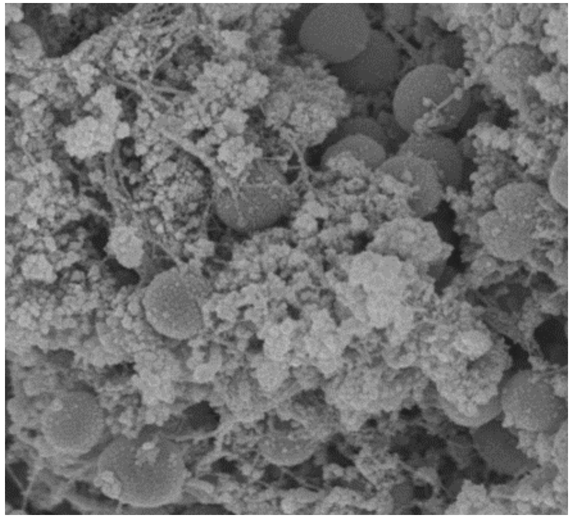

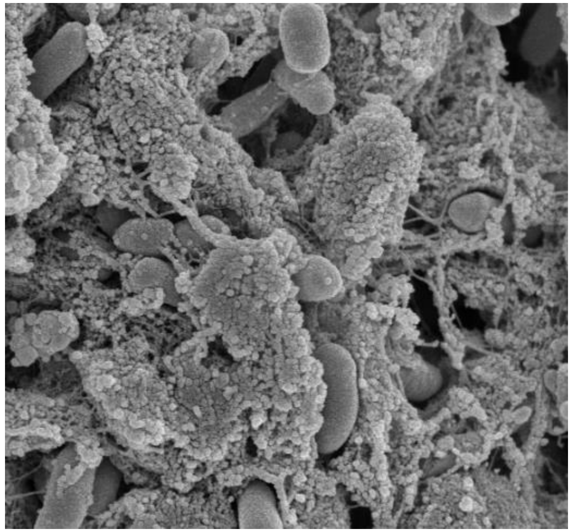

Medium: 2

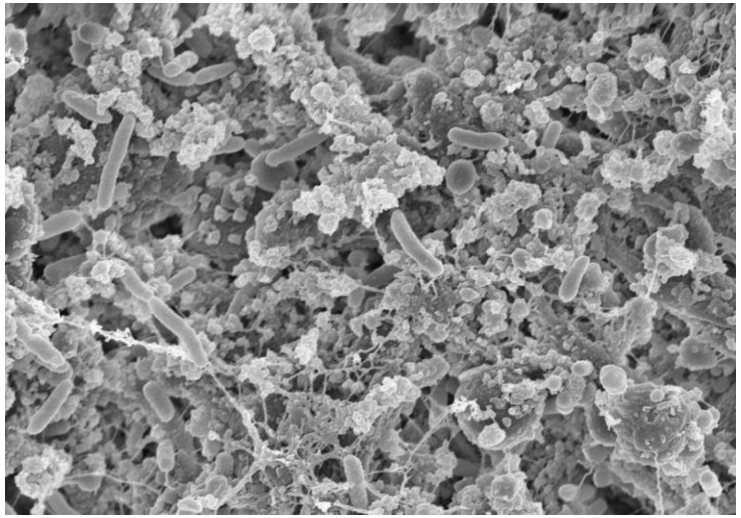

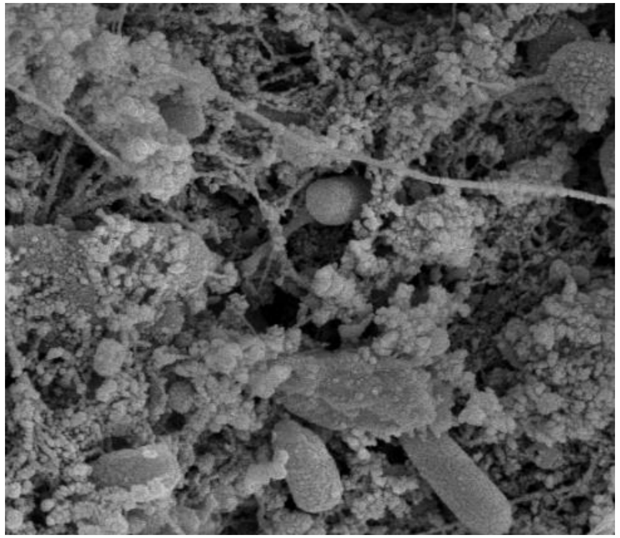

Low: 1

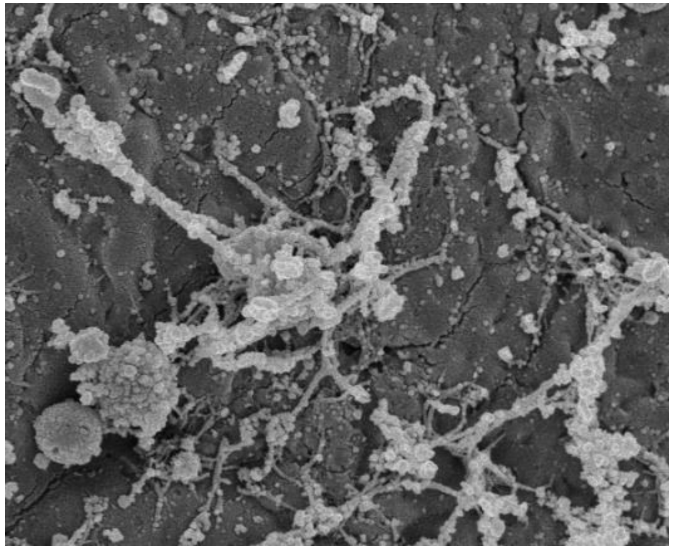

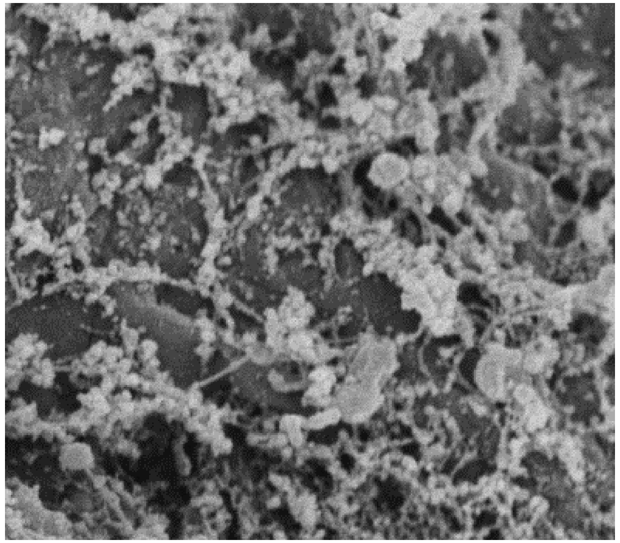

Microbial cell integrity examples

- Shortening
- Compacting

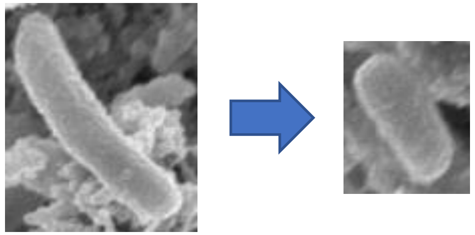

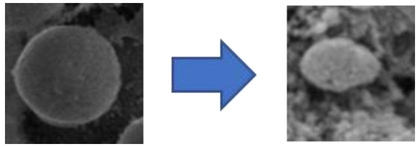

NOT Shortening/Compacting

Appearance of shrinking due to depth of field of view (in comparison to larger-looking cells closer to the surface

- Dimpling

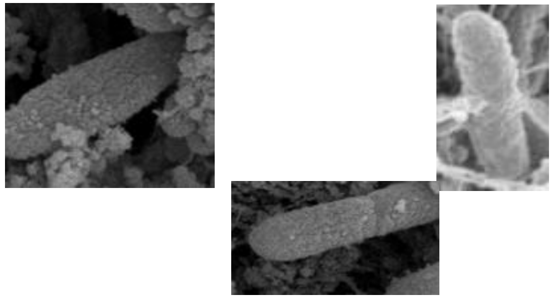

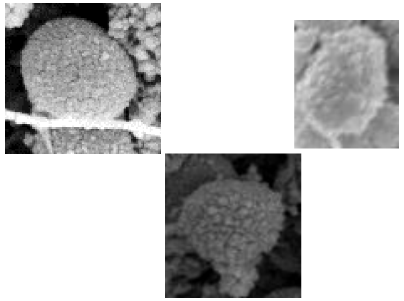

NOT Dimpling/Blistering

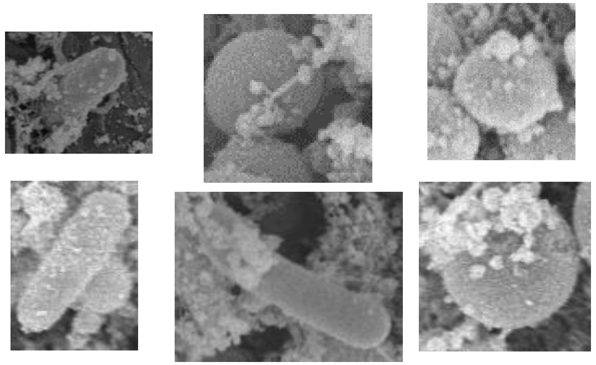

EPS Deposition:
Bright, speckled, not a part of the membrane

- Blistering

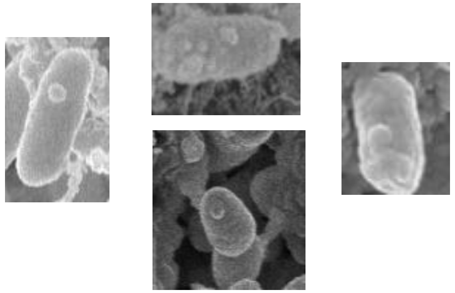

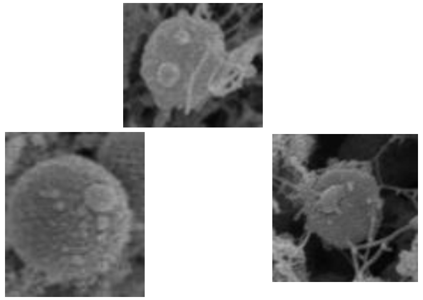

- Active Lysis

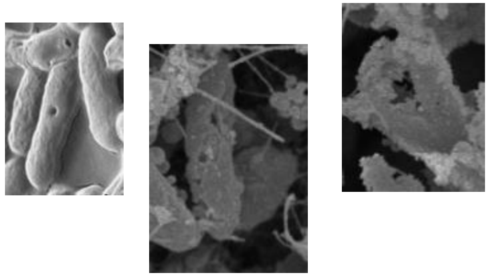

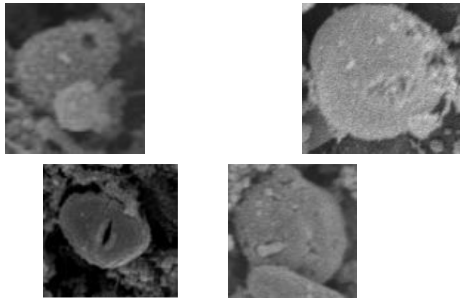

Active lysis is evident when the bacterial membrane breaks down (fractures, holes, fissures)

Microbial cell integrity scoring

- Compacting, dimpling, blistering, lysis evident in:
  - **High (3)**: ≥25% of cells
  - **Medium (2)**: 10-25% of cells
  - **Low: (1)**: ≤10% of cells

Microbial abundance (EPS abundance must be scored first)

- **High (3)**: ≥75 cells/field of view (10,000X mag) in *low-density EPS* **<u>or</u>** cells <u>visibly prevalent throughout</u> *high-density EPS*
- **Medium (2)**: 25-75 cells/field of view (10,000X mag) in *low density EPS* **<u>or</u>** cells prevalently associated with *medium-density EPS* **<u>or</u>** cells sparsely associated with *high-density EPS*
- **Low (1)**: ≤25 cells/field of view (10,000X mag) in *low-density EPS* **<u>or</u>** cells sparsely associated with *medium- or high-density EPS*

Example scoring sheet (General impressions from 3-4 fields of view per sample)

Sample	EPS integrity/abundance	Microbial cell integrity	Microbial abundance
S1	3	3	3
S2	2	3	2
S3	1	2	1
S4	2	1	3
S5	1	1	2
S6	2	1	3
S7	2	2	1
